# Impact of the COVID-19 Pandemic on Violence-Related Emergency Visits: Trend, Seasonality, and Interrupted Time-Series Analysis in Peru, 2015–2024

**DOI:** 10.3390/ijerph22121828

**Published:** 2025-12-05

**Authors:** Claudia Veralucia Saldaña Diaz, Juan Carlos Ezequiel Roque Quezada, Diana Karolina Urbano Sánchez, Víctor Llacsa Saravia, Alberto Gonzales Guzmán

**Affiliations:** 1 Grupo de Investigación NURCARE, Programa Académico de Enfermería, Universidad Privada Norbert Wiener, Lima 00051, Peru; a2021102986@uwiener.edu.pe; 2 Hospital de Emergencias José Casimiro Ulloa, Lima 00051, Peru; juan.roqueq@urp.edu.pe (J.C.E.R.Q.); victor.llacsa@uwiener.edu.pe (V.L.S.); agonzales@hejcu.gob.pe (A.G.G.)

**Keywords:** violence, interpersonal, structural violence, hospital emergency services, COVID-19 pandemic

## Abstract

Violence is a major public health concern, but long-term hospital-based analyses in Latin America remain scarce. This study examined trends, structural breaks, and seasonality of violence-related emergency visits at the José Casimiro Ulloa Emergency Hospital in Lima, Peru, between 2015 and 2024. A retrospective analysis of 14,570 visits was performed, classifying cases according to the World Health Organization typology into self-inflicted, interpersonal community, and interpersonal family violence. Descriptive statistics were stratified by sex, life stage, migratory status, and pandemic period: pre-pandemic (2015–2019), pandemic (2020–2021), and post-pandemic (2022–2024). Time-series analyses included segmented regression and seasonal-trend decomposition. Males (78.3%), youth, and adults were the most affected groups. Interpersonal community violence accounted for 94.2% of cases and closely mirrored the overall series, peaking at 327 visits in July 2019 and falling to 28 in April 2020. Segmented regression showed significant immediate decreases at the onset of the pandemic (−71.6 visits, *p* < 0.001) and partial rebounds after 2022. Family violence remained consistently low and stable, while self-inflicted violence displayed a pre-pandemic upward trend, a sharp decline in 2020, and higher post-pandemic levels. Seasonality was evident before 2020, disappeared during the pandemic, and re-emerged with reduced amplitude thereafter. The COVID-19 pandemic caused abrupt but heterogeneous disruptions in violence-related emergency visits in Lima.

## 1. Introduction

Violence is a major global public health problem, responsible for substantial mortality, disability, and long-term social consequences [1]. The World Health Organization (WHO) categorizes violence into three main forms—self-directed, interpersonal, and collective—providing a framework for monitoring and prevention [2]. In the Region of the Americas, the burden of interpersonal violence is particularly severe, with homicide rates nearly three times higher than the global average [3]. Understanding the epidemiology of violence in specific health-care settings is critical for designing effective interventions.

Emergency departments (EDs) are key entry points for victims of violence and thus serve as a valuable source of data for surveillance and research [4]. Several studies have documented patterns of violence-related visits to EDs worldwide, but evidence from low- and middle-income countries, particularly in Latin America, remains limited [5,6]. In Peru, survey data suggest a high prevalence of interpersonal violence, especially against women [7], yet hospital-based longitudinal studies are scarce. This gap is concerning, as ED data can capture severe and acute cases that may not be reflected in surveys or hotline reports.

The COVID-19 pandemic introduced new challenges, as lockdowns and mobility restrictions not only affected health-care utilization but also altered patterns of violence [8,9]. While some studies reported increases in domestic violence during lockdown [10], others observed decreases in community violence due to reduced public activity [11]. These diverging findings highlight the complexity of interpreting violence trends during crises. Moreover, most prior studies focused on short time frames, without considering long-term trends, seasonality, or structural breaks across pandemic and post-pandemic phases.

Similar dynamics have been reported across Latin America, however the scientific literature on violence during this period is scarce and predominantly focused on gender-based and intimate partner violence, with limited evidence addressing community or self-inflicted forms. Evidence from the region revealed significant under-reporting of domestic violence in Brazil during lockdown [12], and transient increases in domestic and declines in community violence in Colombia and Brazil [13,14]. In Peru, by 2023, the National Institute of Statistics and Informatics (INEI) reported that more than 50% of women aged 15–49 had experienced some form of violence perpetrated by their partner [15]. Projections for Brazil suggest a concerning scenario, with estimates indicating a potential 95% increase in violence against women over the next decade [16]. Meanwhile, violence in Peru has shown a worrying upward trend, with around 500 homicides recorded every quarter during 2024 and 2025, reflecting the intensification of public insecurity nationwide [17]. In this context, examining how violence-related cases evolve within hospital emergency services becomes increasingly relevant to better understand the dynamics and health impact of this growing social problem in the region.

To address these gaps, we analyzed ten years (2015–2024) of violence-related visits at a major emergency hospital in Lima, Peru. Using the WHO typology, cases were classified into self-inflicted, interpersonal community, and interpersonal family violence. We combined descriptive statistics, stratified time-series analysis, segmented regression to identify structural breaks during and after the COVID-19 pandemic, and seasonal-trend decomposition to evaluate recurrent patterns. Our findings provide new insights into how violence-related emergencies evolved before, during, and after the pandemic, with implications for surveillance and prevention strategies in similar urban contexts.

## 2. Materials and Methods

Study Design and Setting

This is a retrospective observational study based on institutional records of violence-related visits at the José Casimiro Ulloa Emergency Hospital (HEJCU) in Lima, Peru, covering the period January 2015 to December 2024. HEJCU is a high-complexity public referral hospital located in Lima, Peru. HEJCU specializes in the provision of emergency and acute care services and functions as a national reference center for trauma, violence-related injuries, and other urgent medical conditions. The hospital serves the general public and primarily attends low-income and uninsured populations from Lima and surrounding provinces, as it is a public emergency facility open to all individuals requiring urgent care, regardless of socioeconomic status or geographic origin. The analysis aimed to describe the epidemiological characteristics of these visits and to examine temporal patterns, including trends, seasonality, and structural breaks associated with the COVID-19 pandemic.

Data Collection and Variables

The dataset consisted of routinely collected emergency department records, including the date of admission, sex, age, type of document presented at admission, and reason for consultation (motive of violence-related care). The hospital’s Office of Statistics and Informatics provided the research team with the complete list of “reason for visit” categories, from which three investigators of this study—each with expertise in emergency medicine and epidemiological research—independently reviewed and selected those corresponding to violence-related care. Their selections were subsequently discussed in joint sessions until a consensus was reached, ensuring consistent and unbiased case identification.

Sociodemographic variables included sex (female, male), life stage, classified into child (0–11 years), adolescent (12–17 years), youth (18–29 years), adult (30–59 years), and elderly (≥60 years), and migratory status. For the latter, document categories were collapsed into two groups: foreigners (foreign documentation, foreign identification card, passport, were assigned to this category) and natives (including national ID, national auto-generated temporary ID codes, newborn codes, and national military/police codes). Missing information was excluded from the final dataset; however, these cases represented less than 5% of the total and did not affect the overall analysis.

The type of violence was reorganized into three analytical categories following the World Health Organization (WHO) typology of violence [2]. (1) Self-inflicted violence included all cases coded as self-harm or auto-aggression. (2) Interpersonal community violence grouped physical assault, robbery with assault, gunshot injuries, stabbing, robbery with firearm, sexual assault, rape, kidnapping, and referred sexual or physical violence. (3) Interpersonal family violence included all cases recorded as domestic or intrafamilial violence. This WHO-based harmonization allowed for consistency and comparability across the study period. Although the HEJCU has no assigned jurisdiction and is open to patients from across Peru, it predominantly serves low-income populations from the central–southern area of Lima, with approximately 97% of patients covered by the *Seguro Integral de Salud* (SIS), a national insurance program for individuals of limited financial resources.

Definition of Periods

For analytical purposes, three timeframes were defined: pre-pandemic (2015–2019), pandemic (2020–2021), and post-pandemic (2022–2024). The years 2020 and 2021 were selected to represent the pandemic period because they coincided with the lockdown measures implemented in Peru, during which mobility restrictions and health service reorganizations strongly influenced emergency care utilization patterns.

Descriptive Analysis

Absolute and relative frequencies were calculated for categorical variables, which were compared across the three timeframes using the Pearson chi-square test. Results were summarized in Table 1, where counts and percentages are presented as n (%). A significance level of *p* < 0.05 was adopted for all hypothesis testing.

Time Series Analysis

For temporal analyses, data were aggregated into monthly counts of visits. Initially, the general series was analyzed, followed by stratifications by sex, life stage, and type of violence. Graphical exploration included quarterly background shading and highlighting of the pandemic years (2020–2021) to facilitate visualization of structural breaks.

Segmented Regression

To assess the impact of the COVID-19 pandemic as a structural break, segmented regression models (interrupted time series, ITS) were fitted. Two interruption points were defined a priori: January 2020 (onset of pandemic/lockdown) and January 2022 (post-pandemic period).

The segmented regression model was specified as follows:

*Yt* = *β*0 + *β*1*⋅t* + *β*2*⋅D*1*t* + *β*3*⋅tD*1*t* + *β*4*⋅D*2*t* + *β*5*⋅tD*2*t* + *ϵt*

where

- *Yt* = monthly number of visits at time *t*.
- *t* = continuous time in months from study start.
- *D*1*t* = indicator variable for the pandemic period (1 if *t* ≥ Jan 2020, 0 otherwise).
- *tD*1*t* = interaction term (time since start of pandemic).
- *D*2*t* = indicator variable for the post-pandemic period (1 if *t* ≥ Jan 2022, 0 otherwise).
- *tD*2*t* = interaction term (time since start of post-pandemic).

This allowed estimation of immediate level changes (*β*2, *β*4) and slope changes (*β*3, *β*5) between periods.

To address serial autocorrelation and heteroskedasticity common in monthly count data, Newey–West (HAC) robust standard errors with 12 lags were used. Model fit was evaluated graphically by overlaying predicted values on observed data.

Seasonal-Trend Decomposition

To explore seasonality, the monthly series was decomposed using Seasonal-Trend decomposition based on Loess (STL) with a periodicity of 12 months. The STL method separates the series into: (1) long-term trend, (2) recurring seasonal pattern, and (3) residuals. Residuals were displayed as scatter plots to emphasize irregular fluctuations and outliers. The pandemic period was highlighted in all graphs to illustrate disruption of seasonal dynamics. STL was applied both to the general series and separately for each category of violence.

Software

All analyses were conducted using Python version 3.11. The pandas library was used for data management, statsmodels for regression modeling, and matplotlib for data visualization. STL decomposition was performed using the seasonal module from statsmodels.tsa.seasonal.

Ethical Considerations

This study utilized anonymized secondary data obtained from hospital administrative records. The Ethics Committee of the José Casimiro Ulloa Emergency Hospital waived the requirement for ethical approval, as the analysis was based solely on secondary data. The study was conducted in accordance with the principles of the Declaration of Helsinki.

## 3. Results

We analyzed a total of 14,570 violence-related visits recorded at the José Casimiro Ulloa Emergency Hospital between 2015 and 2024. Results are presented as descriptive characteristics, temporal trends, segmented regression, and seasonality analyses.

### 3.1. Descriptive Characteristics

Table 1 summarizes the sociodemographic and clinical characteristics of violence-related visits across three time periods: pre-pandemic (2015–2019), pandemic (2020–2021), and post-pandemic (2022–2024). Males accounted for the majority of cases in all periods (78.3% overall). Youth (18–29 years) and adults (30–59 years) represented the largest age groups, while elderly and those under 18 years each represent less than 5% of visits. Regarding migratory status, foreigners represented 17.5% of cases in the pre-pandemic period but decreased to 6.6% during the pandemic and remained stable at 6.7% in the post-pandemic period. Interpersonal community violence was the most frequent type across all periods (94.2% overall), followed by interpersonal family violence (3.3%) and self-inflicted violence (2.4%). The street was the most common place of assault (69.6%), followed by the home (25.4%). Significant differences were observed across periods for all variables (*p* < 0.05).

### 3.2. Temporal Trends

#### 3.2.1. Overall Series

**Figure 1:**
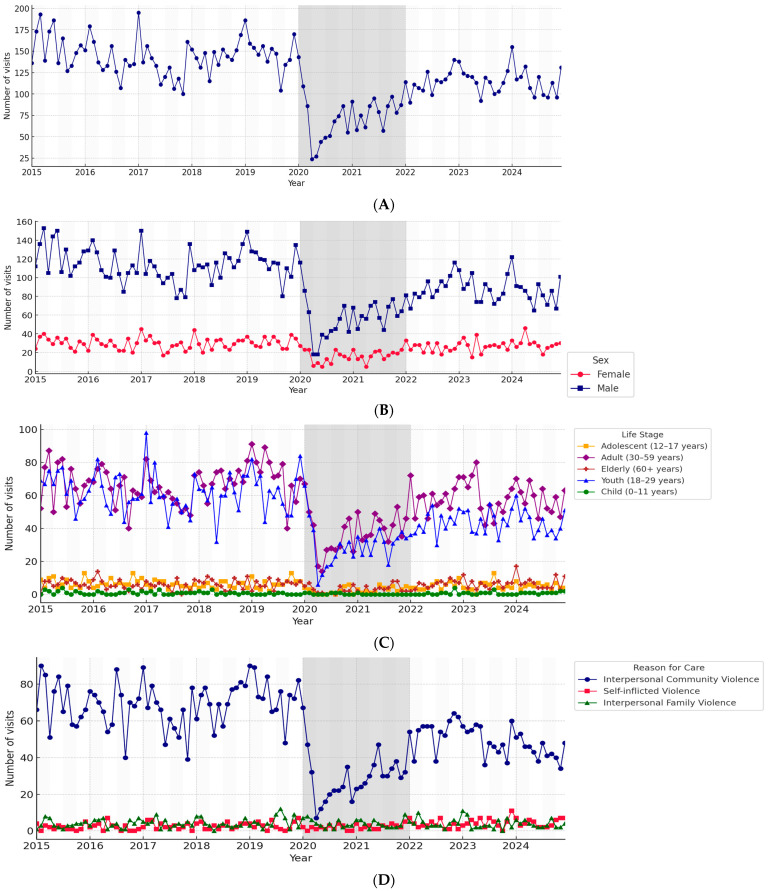
Time series of violence-related emergency visits at José Casimiro Ulloa Emergency Hospital, 2015–2024: (**A**) total visits, (**B**) by sex, (**C**) by life stage, and (**D**) by reason for care. Light gray and white bands denote quarterly intervals; the dark gray band highlights the COVID-19 pandemic period (2020–2021).

Figure 1A shows the monthly time series of all violence-related visits from 2015 to 2024. The mean monthly number of visits was 121.4, with a maximum of 327 cases recorded in July 2019 and a minimum of 28 cases in April 2020, the latter coinciding with the strictest lockdown restrictions. A marked decline was observed at the beginning of 2020, coinciding with the onset of the COVID-19 pandemic, followed by a gradual recovery in subsequent years that stabilized at lower levels than those observed before the pandemic.

#### 3.2.2. Stratification by Sex

When stratified by sex (Figure 1B), both males and females exhibited a parallel decline in early 2020. Male patients consistently accounted for the majority of visits, averaging 95.0 monthly cases (range: 22–246), while females averaged 26.4 monthly cases (range: 4–81). Despite lower absolute counts, the female series displayed a relatively higher proportional increase after 2022, rising from 20.7% of cases pre-pandemic to 23.7% post-pandemic.

#### 3.2.3. Stratification by Life Stage

As shown in Figure 1C, youth (18–29 years) and adults (30–59 years) consistently contributed the highest number of visits. Youth accounted for a monthly average of 50.3 cases (range: 10–141), while adults averaged 58.6 cases (range: 12–160). Children (0–11 years) and adolescents (12–17 years) contributed much smaller proportions, averaging 0.7% and 4.4% of cases, respectively. Elderly cases remained stable at very low levels throughout the study period, representing only 4.7% overall.

#### 3.2.4. Stratification by Type of Violence

Figure 1D illustrates temporal trends by type of violence. Interpersonal community violence closely mirrored the overall series, averaging 114.4 monthly cases (range: 25–309), with a sharp pandemic-related decline and partial recovery post-2022. Interpersonal family violence accounted for a much smaller burden, averaging 4.2 monthly cases (range: 0–17), while self-inflicted violence averaged 2.9 monthly cases (range: 0–15). Both of these categories showed no evident structural breaks, but greater variability was noted in isolated months, particularly after 2022.

### 3.3. Segmented Regression Analysis

#### 3.3.1. Overall Series

**Figure 2:**
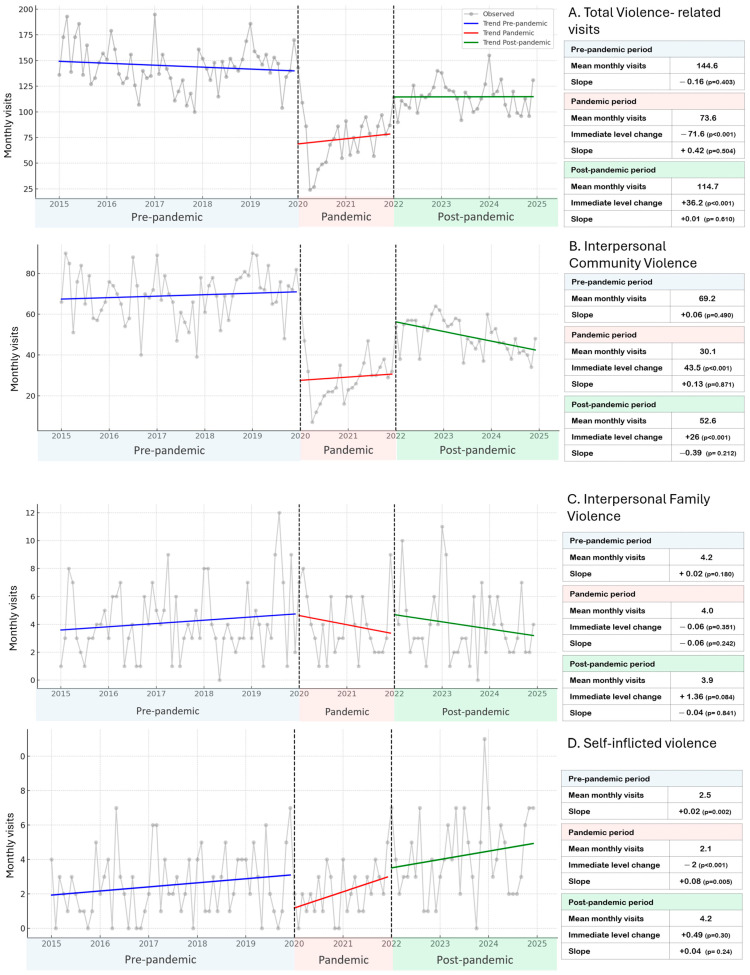
Segmented time-series trends of violence-related visits at the José Casimiro Ulloa Emergency Hospital, 2015–2024: (**A**) total visits, (**B**) of interpersonal community violence, (**C**) Of interpersonal familiar violence, and (**D**) of self-inflicted violence. The gray dots represent observed monthly counts. Fitted segmented regression lines are shown in blue for the pre-pandemic period (2015–2019), red for the pandemic period (2020–2021), and green for the post-pandemic period (2022–2024). Vertical dashed lines indicate the onset of the COVID-19 pandemic (January 2020) and the beginning of the post-pandemic period (January 2022).

Figure 2A presents the segmented regression of overall monthly visits. The mean number of monthly visits decreased from 144.6 pre-pandemic to 73.6 during the pandemic, before partially recovering to 114.7 post-pandemic. At the onset of the pandemic in January 2020, there was an immediate level decrease of –71.6 visits (*p* < 0.001). At the beginning of the post-pandemic period in January 2022, a partial rebound of +36.2 visits (*p* < 0.001) was observed. The estimated slopes were not statistically significant across all periods (pre-pandemic: −0.16 visits/month, *p* = 0.40; pandemic: +0.42 visits/month, *p* = 0.50; post-pandemic: +0.01 visits/month, *p* = 0.61), indicating that the pandemic primarily altered the level of visits rather than the underlying monthly trend.

#### 3.3.2. Interpersonal Community Violence

Interpersonal community violence followed a similar dynamic to the overall series. The mean monthly number of visits fell from 69.2 pre-pandemic to 30.1 during the pandemic, with a subsequent increase to 52.6 post-pandemic. The onset of the pandemic was associated with an immediate level decrease of −43.5 visits (*p* < 0.001), while the transition to the post-pandemic period showed a positive level change of +26.0 visits (*p* < 0.001). Slopes were non-significant in all periods (pre-pandemic: +0.06, *p* = 0.49; pandemic: +0.13, *p* = 0.87; post-pandemic: −0.39, *p* = 0.21), suggesting that fluctuations were mainly explained by abrupt changes in level rather than gradual trends. (Figure 2B).

#### 3.3.3. Interpersonal Family Violence

For interpersonal family violence (Figure 2C), mean monthly visits remained consistently low (≈4.2 pre-pandemic, 4.0 during the pandemic, and 3.9 post-pandemic). No significant immediate level changes were detected at either breakpoint (pandemic onset: –0.06, not significant; post-pandemic onset: +1.36, *p* = 0.08). Slopes were also non-significant across all periods (pre-pandemic: +0.02, *p* = 0.18; pandemic: −0.06, *p* = 0.24; post-pandemic: −0.04, *p* = 0.84). The model explained virtually no variance (adjusted R^2^ ≈ 0), reflecting the stability and low frequency of this category.

#### 3.3.4. Self-Inflicted Violence

Self-inflicted violence displayed a distinct pattern. The mean monthly visits were 2.5 pre-pandemic, slightly decreased to 2.1 during the pandemic, and increased to 4.2 post-pandemic. At the pandemic onset, there was a sharp immediate level decrease of −2.0 visits (*p* < 0.001), followed by a significant recovery during the pandemic period (slope = +0.08 visits/month, *p* = 0.005). The pre-pandemic slope was small but statistically significant (+0.02, *p* = 0.002), indicating a steady upward trend before 2020. In the post-pandemic period, the level change was modest and not significant (+0.49, *p* = 0.30), with a stable slope (+0.04, *p* = 0.24). This suggests that self-inflicted violence consolidated at a slightly higher post-pandemic level, without a clear long-term trend.

### 3.4. Seasonality Analysis

#### 3.4.1. Overall Series Decomposition

**Figure 3:**
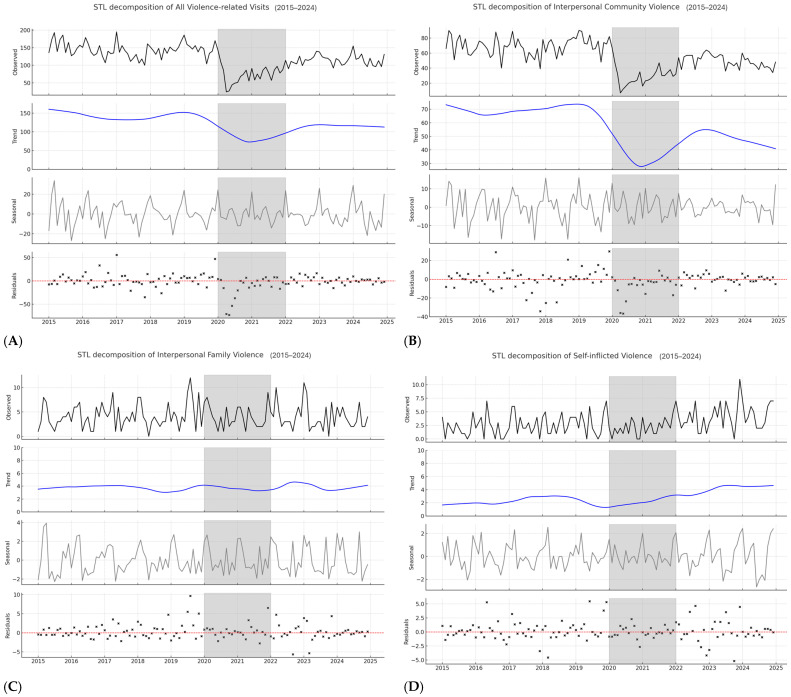
Seasonal-Trend decomposition of monthly interpersonal community violence visits at the José Casimiro Ulloa Emergency Hospital, 2015–2024: (**A**) total visits, (**B**) of interpersonal community violence, (**C**) Of interpersonal familiar violence, and (**D**) of self-inflicted violence. The series was decomposed using Seasonal-Trend decomposition via Loess (STL). The top panel displays the observed monthly counts (black solid line), with the COVID-19 pandemic period (2020–2021) highlighted in gray. The second panel shows the estimated long-term trend (blue solid line). The third panel represents the recurrent seasonal component (gray line), and the bottom panel shows the residuals (black crosses), with the red dashed line indicating the zero baseline (mean of residuals).

The STL decomposition of the overall series (Figure 3A) showed a clear annual seasonal pattern prior to 2020, with regular peaks and troughs across the calendar year. This seasonality nearly disappeared during the pandemic period (2020–2021), when mobility restrictions disrupted normal temporal patterns. After 2022, the seasonal component partially re-emerged, although with reduced amplitude compared to the pre-pandemic years.

#### 3.4.2. Interpersonal Community Violence Decomposition

For interpersonal community violence (Figure 3B), the seasonal pattern was strong and consistent before 2020, with pronounced monthly oscillations. This regularity was suppressed during the pandemic, when the seasonal component flattened. A weaker seasonal cycle reappeared after 2022, though with lower intensity than in the pre-pandemic period.

#### 3.4.3. Interpersonal Family Violence Decomposition

The decomposition of interpersonal family violence (Figure 3C) revealed only mild oscillations, without a clearly defined annual seasonal component. The pattern remained essentially flat and weak throughout all three periods, reflecting the stability of this low-frequency category.

#### 3.4.4. Self-Inflicted Violence Decomposition

Self-inflicted violence (Figure 3D) presented an irregular and unstable seasonal component. While some annual oscillations were detectable, they were small in magnitude and inconsistent across the study period. After 2022, the seasonal component became more variable, reflecting increased irregularity in monthly patterns.

## 4. Discussion

This ten-year time-series analysis of violence-related emergency visits at a referral hospital in Lima, Peru, revealed three major findings. First, overall and interpersonal community violence-related visits exhibited a sharp decline at the onset of the COVID-19 pandemic, followed by partial recovery after 2022. Second, interpersonal family violence remained stable at low frequencies throughout the study period, while self-inflicted violence displayed a distinct dynamic, with a pre-pandemic upward trend, a sharp drop in early 2020, and a subsequent rebound that stabilized at higher post-pandemic levels. Third, seasonality was evident before 2020 but disappeared during the pandemic and only partially re-emerged with reduced amplitude in the post-pandemic years. These results indicate that the pandemic produced abrupt structural breaks in violence dynamics, with heterogeneous effects depending on the type of violence.

The predominance of males and young adults among cases is consistent with global and regional evidence highlighting the disproportionate burden of interpersonal violence in these groups [3,18]. In Latin America, young men are at particularly high risk due to structural inequalities, high rates of community violence, and limited access to preventive interventions [19,20]. The significant decrease in visits by foreigners during 2020–2021 can be explained by Peru’s stringent border closures and mobility restrictions [21], echoing similar patterns reported in other contexts where migration flows were drastically reduced [22].

The immediate drop in community violence-related visits during the pandemic mirrors findings from studies in Peru and abroad. Calderon-Anyosa and Kaufman [11] documented reductions in homicide and traffic deaths during Peru’s national lockdown, while Mohler et al. [23] and Leslie and Wilson [24] reported decreased crime in US cities under social distancing mandates. These reductions have been attributed to limited mobility, closure of entertainment venues, and reductions in alcohol consumption [25]. However, our results also show that these changes were temporary, as levels increased again once restrictions eased, supporting the notion that lockdown effects represented short-term disruptions rather than structural trend shifts [26,27].

Comparatively, studies from other Latin American countries have reported similar trends. In Chile, a temporary reduction in homicides and assaults was observed during confinement, followed by post-pandemic rebounds [28]. In Brazil, analyses of hospital admissions indicated underreporting of domestic violence cases during lockdown [29], while in Colombia, police reports showed a transient increase in household violence followed by a decline in community-related incidents [13]. These parallels suggest that, across the region, violence patterns were shaped not only by restrictions but also by the resilience and limitations of national surveillance systems.

Conversely, interpersonal family violence showed no significant increase in emergency visits, despite hotline and survey data suggesting increases in domestic violence during confinement in Peru and other countries [14,30,31]. Hernández-Vásquez et al. [10] demonstrated rises in calls to Peru’s Línea 100 helpline, and global reports indicated that stay-at-home orders intensified risks of partner violence [32,33]. The absence of change in hospital visits may reflect reduced care-seeking during lockdown, underreporting, or a mismatch between perceived violence and clinically attended injuries. Similar discrepancies between administrative health data and hotline reports have been described in Brazil [34] and Mexico [35], reinforcing the need for triangulated surveillance systems.

Self-inflicted violence presented a different trajectory. The significant upward trend before 2020 is consistent with global increases in self-harm presentations among youth and young adults [36,37]. The sudden decline in early 2020 parallels reports from England, the US, and Japan, where suicide and self-harm presentations initially fell during the first months of the pandemic [38,39]. However, these declines have been interpreted as artifacts of reduced health-care access rather than true decreases in incidence [40]. The rebound and stabilization at higher levels post-pandemic observed in our data aligns with findings of increased psychological distress and suicidality after prolonged lockdowns [41]. Importantly, our observation of greater variability in the post-2022 period is consistent with the hypothesis that the pandemic introduced long-lasting volatility in self-harm risk patterns [42].

Seasonality analyses provide further insights. The disappearance of regular seasonal oscillations during 2020–2021 reflects the disruption of social and environmental drivers of violence, such as holidays, festivities, and climatic conditions [43,44]. Previous studies in Europe reported that analyses have linked temperature and daylight hours to fluctuations in assaults [45]. Our finding that seasonal patterns only partially re-emerged post-2022 suggests that pandemic-related social changes may have permanently attenuated or reshaped these dynamics, a phenomenon also noted in studies of trauma admissions in Spain [46].

Beyond these temporal and demographic patterns, the observed differences among types of violence can be better understood through broader social and structural determinants. The structural break in violence-related emergency visits during the pandemic was likely driven primarily by the sharp decline in interpersonal community violence, as strict mobility restrictions drastically limited opportunities for robberies, assaults, and other street conflicts. The concurrent reduction in alcohol and drug availability and consumption in public spaces may also have contributed to this temporary decrease. In contrast, intrafamilial and self-inflicted violence likely increased during confinement, fueled by household stress, prolonged isolation, illness or death of family members, and widespread job losses. Nevertheless, fear of contagion and limited hospital access may have reduced care-seeking for these causes, so only the most severe cases were likely to present to emergency services.

Taken together, our results situate Peru within a broader global narrative: while community violence was transiently suppressed by lockdown measures, domestic violence and self-harm followed more complex patterns, influenced by both access to care and underlying social determinants. These findings emphasize the need to interpret health-service data in light of complementary sources and to consider both abrupt shocks and cyclical processes when analyzing violence dynamics. It is also important to note that the available database did not include information on alcohol or drug use at the time of admission, which represents a limitation that may restrict the interpretation of behavioral or contextual risk factors underlying these patterns.

From a policy perspective, these findings underscore the urgent need to strengthen hospital-based surveillance systems for violence and ensure their integration with intersectoral prevention strategies. In the current context of escalating violence in Peru—with more than 500 homicides per trimester reported nationwide since 2024 [17] and with approximately 67,000 cases handled by the Women’s Emergency Centers, a state-run support and protection services for victims of violence [47]—it is crucial to implement standardized hospital registries that record the specific reason for visit rather than relying solely on ICD-10 codes. Such detailed registries would provide a clearer picture of the health and economic burden of violence, strengthen early detection systems, and support coordinated prevention strategies across health, law enforcement, and social sectors.

## 5. Conclusions

This ten-year analysis demonstrates that the COVID-19 pandemic represented a structural break in violence-related emergency visits, producing sharp but heterogeneous disruptions across different types of violence. Drawing on a large, decade-long hospital dataset and applying advanced analytical methods such as segmented regression and seasonal decomposition, this study offers a more comprehensive understanding of temporal patterns than descriptive approaches alone. Overall, the findings highlight the critical need to strengthen hospital information and surveillance systems to generate timely, high-quality data capable of guiding prevention strategies and improving preparedness for future public health crises.

## Figures and Tables

**Table 1:** Distribution of Characteristics by Period: Pre-pandemic (2015–2019), Pandemic (2020–2021), and Post-pandemic of violence related visits in (2022–2024); gray = subanalysis.

Variable	Pre-Pandemic (2015–2019)	Pandemic (2020–2021)	Post-Pandemic (2022–2024)	Total	Valor *p*
**Total**	8676	1766	4128	14,570	
**Sex**					<0.001 *
Female	1797 (20.7%)	392 (22.2%)	979 (23.7%)	3168 (21.7%)	
Male	6879 (79.3%)	1374 (77.8%)	3149 (76.3%)	11,402 (78.3%)	
**Life Stages**					<0.001 *
0–11 years	52 (0.6%)	6 (0.3%)	27 (0.7%)	85 (0.6%)	
12–17 years	396 (4.6%)	59 (3.3%)	185 (4.5%)	640 (4.4%)	
18–29 years	3743 (43.6%)	717 (40.6%)	1574 (38.1%)	6034 (41.7%)	
30–59 years	4021 (46.8%)	910 (51.5%)	2106 (51.0%)	7037 (48.6%)	
60+ years	371 (4.3%)	74 (4.2%)	236 (5.7%)	681 (4.7%)	
**Migratory status**					<0.001 *
Foreigner	1519 (17.51%)	121 (6.58%)	263 (6.37%)	1903 (13.06%)	
Native	7157 (82.49%)	1645 (93.15%)	3865 (93.63%)	12,667 (86.94%)	
**Type of Violence**					<0.001 *
Self-inflicted	151 (1.74%)	50 (2.83%)	152 (3.68%)	353 (2.42%)	
Interpersonal: Community	8275 (95.37%)	1620 (91.73%)	3834 (93.62%)	13,729 (94.22%)	
Physical assault	3845 (46.5%)	794 (49.0%)	1858 (48.5%)	6497 (47.3%)	
Robbery with assault	3702 (44.7%)	586 (36.2%)	1492 (38.9%)	5780 (42.1%)	
Gunshot injury	420 (5.1%)	99 (6.1%)	223 (5.8%)	742 (5.4%)	
Stabbing	161 (1.9%)	83 (5.1%)	134 (3.5%)	378 (2.8%)	
Robbery with gunshot	114 (1.4%)	44 (2.7%)	65 (1.7%)	223 (1.6%)	
Sexual assault	21 (0.3%)	9 (0.6%)	37 (1.0%)	67 (0.5%)	
Rape	9 (0.1%)	5 (0.3%)	24 (0.6%)	38 (0.3%)	
Kidnapping	3 (0.0%)	0 (0.0%)	1 (0.0%)	4 (0.0%)	
Interpersonal: Familiar	250 (2.88%)	96 (5.43%)	142 (3.43%)	488 (3.34%)	
**Place of assault**					<0.001 *
Street	6063 (67.6%)	1213 (68.7%)	2868 (69.5%)	10,144 (69.63%)	
Home	2198 (24.9%)	481 (27.2%)	1022 (24.7%)	3701 (25.40%)	
Workplace	170 (2.0%)	29 (1.6%)	81 (2.0%)	280 (1.92%)	
Recreational center	143 (1.6%)	7 (0.4%)	66 (1.6%)	216 (1.48%)	
Police Station	33 (0.4%)	31 (1.8%)	65 (1.6%)	129 (0.89%)	
Airport	36 (0.4%)	3 (0.2%)	10 (0.2%)	49 (0.34%)	
Public transportation	27 (0.3%)	1 (0.1%)	7 (0.2%)	35 (0.24%)	
Province	6 (0.1%)	1 (0.1%)	8 (0.2%)	15 (0.10%)	
**Type of discharge**					<0.001 *
Deceased	46 (0.5%)	10 (0.6%)	22 (0.5%)	51 (0.4%)	
Discharged by medical indication	8209 (94.6%)	1654 (93.7%)	3741 (90.6%)	13,313 (91.4%)	
Voluntary discharge	420 (4.8%)	100 (5.7%)	365 (8.8%)	318 (2.2%)	
Referral/transfer	1 (0%)	2 (0.1%)	0 (0%)	3 (0.0%)	

* *p* < 0.005.

## Data Availability

The data that support the findings of this study were obtained from the anonymized institutional database of the José Casimiro Ulloa Emergency Hospital. Due to privacy and ethical restrictions, these data are not publicly available. De-identified data may be made available from the corresponding author upon reasonable request and with permission of the hospital administration.

## Abbreviation

The following abbreviation is used in this manuscript:

HEJCU	Hospital de Emergencias Jose Casimiro Ulloa
